# Is It Just a Marker for Increased Care?

**DOI:** 10.1371/journal.pmed.0030406

**Published:** 2006-09-26

**Authors:** Richard Hockey

Because of the nature of the analysis used in this study [1], no conclusion is possible. There are plenty of examples in the literature demonstrating the “ecological fallacy”. Studies such as this have very little utility other than to generate hypotheses. I tend to think that this association is a marker for greater recognition and treatment for depression. However, it’s a brave epidemiologist who would draw any conclusions at all from an ecological association such as this where the outcome is relatively rare.
